# Betaine Alleviates High-Fat Diet Induced Excessive Lipid Deposition in Gibel Carp Hepatopancreas and L8824 Cells by Enhancing VLDL Secretion through HNF4*α*/MTTP Pathway

**DOI:** 10.1155/2024/8886237

**Published:** 2024-03-04

**Authors:** Xiaojing Dong, Jianqiao Wang, Mengjie Zhao, Xuedi Du, Hongying Fan, Yuanyuan Fu, Zhiyuan Gong, Shuyan Miao

**Affiliations:** ^1^College of Animal Science and Technology, Yangzhou University, Yangzhou, Jiangsu, China; ^2^Department of Biological Sciences, National University of Singapore, Singapore, Singapore; ^3^Ningbo Institute of Oceanography, Ningbo 315832, Zhejiang, China

## Abstract

Betaine, a methyl donor, plays a crucial role in lipid metabolism. Previous studies have shown that appropriate betaine supplementation in a high-fat diet reduces triglycerides (TG) of serum and hepatopancreas in fish. However, the underlying mechanism remains unclear. This study examined whether betaine can enhance the secretion of very low-density lipoprotein (VLDL) and sought to identify the specific mechanisms through which this enhancement occurs. A lipid accumulation model was established in gibel carp and L8824 cells using a high-fat diet and oleic acid, respectively. Different doses of betaine (1, 4, and 16 g/kg in the diet; 400 *μ*mol in cell culture) were administered, and measurements were taken for lipid deposition, gene expression of HNF4*α*, MTTP, and ApoB, as well as the regulation of *Mttp* and *Apob* promoters by HNF4*α*. The results showed that betaine supplementation mitigated lipid droplet accumulation, TG levels, and VLDL production induced by the high-fat diet in gibel carp hepatopancreas and L8824 cells. Moreover, betaine not only increased VLDL content in the cell culture supernatant but also reversed the inhibitory effects of the high-fat diet on protein expression of MTTP, ApoB, and HNF4*α* in both gibel carp hepatopancreas and L8824 cells. Additionally, HNF4*α* exhibits transactivating activity on the promoter of *Mttp* in gibel carp. These findings suggest that betaine supplementation exerts its effects through the HNF4*α*/MTTP/ApoB pathway, promoting the assembly and secretion of VLDL and effectively reducing lipid accumulation in the hepatopancreas of farmed gibel carp fed a high-fat diet.

## 1. Introduction

Dietary lipids play a crucial role in the growth and development of fish, providing energy and essential fatty acids necessary for their overall health [1]. In recent years, high-fat diets have become increasingly popular in intensive aquaculture as they offer increased energy, promoting protein sparing, and enhancing fish growth [2–4]. However, high-fat diets also present potential challenges in fish farming.

One challenge is the increased susceptibility of high-fat diets to oxidation during storage, primarily due to their higher unsaturated fatty acid content. Oxidized oil can be toxic to fish, posing a risk to their health and well-being [5]. Another challenge associated with high-fat diets is the induction of hepatic steatosis, characterized by the excessive deposition of lipids in the liver [2]. While short-term feeding of high-fat diets (<6 weeks) may promote fish growth, long-term exposure can disrupt metabolic processes and result in adverse effects on fish health [6–8]. Therefore, further research and optimization of feeding practices are necessary to ensure sustainable and healthy aquaculture.

Betaine, also known as trimethylglycine, is naturally present in animals, plants, and microorganisms, serves various physiological functions as a methyl group donor. Extensive research on animals has highlighted the significant nutritional and physiological roles of betaine, including promoting growth, exerting antistress effects, improving reproductive performance, acting as an antioxidant, and providing osmotic protection [9, 10]. Studies conducted in fish have suggested that betaine supplementation can effectively reduce lipid deposition [11]. However, the precise mechanisms underlying this phenomenon are not yet fully understood and require further investigation.

We previously observed that appropriate supplementation of betaine in a basic diet had a beneficial effect on alleviating lipid metabolism disorders induced by high-fat feeding, including a reduction in triglyceride (TG) levels in hepatopancreas [12]. In humans, the secretion of TG in the liver depends on the production and release of very low-density lipoprotein (VLDL), relying on the coordinated function of several genes, such as *Mttp* and *Apob*, which encode microsomal triglyceride transfer protein (MTTP) and apolipoprotein B-100 (ApoB-100), respectively. MTTP and ApoB-100 play crucial roles in the assembly and secretion of VLDL [13, 14]. Unlike in mammals, fish ApoB only encodes a full-length protein similar to human ApoB-100, probably due to the shortage of the apolipoprotein B mRNA editing enzyme catalytic subunit 1 [15, 16].

Hepatocyte nuclear factor 4*α* (HNF4*α*) is a nuclear hormone receptor that serves as a critical regulator of gene expression in the liver. It plays a vital role in controlling the basal expression of numerous genes essential for various metabolic pathways, including those related to bile acid, lipid, glucose, and drug metabolism [17, 18]. Studies conducted in adult mice have demonstrated the significance of HNF4*α* in hepatic lipid metabolism. For example, liver-specific conditional knockout of *Hnf4α* in mice resulted in severe steatosis, which was associated with disrupted secretion of VLDL and dysregulation of ApoB and MTTP expression [19–21]. While the functions of HNF4*α*, MTTP, and ApoB in maintaining hepatic lipid homeostasis in mammals are well determined, their specific roles in fish lipid metabolism, particularly in the alleviation of lipid deposition induced by dietary betaine, have yet to be fully understood.

In this study, we analyzed the effect of betaine on lipid deposition in the hepatopancreas of gibel carp and L8824 cells, as well as on the gene expression of HNF4*α*, MTTP, and ApoB at the transcription and protein levels. Additionally, a dual-luciferase reporter assay was employed to examine the potential role of HNF4*α* in *Mttp* and *Apob* transcription. The findings from this study will contribute to elucidating the molecular mechanisms underlying the effects of betaine on lipid metabolism in fish, providing valuable insights for further research in this field.

## 2. Materials and Methods

### 2.1. Experimental Diets, Feeding Trials, and Sample Collection

The formulation of experimental diets, feeding protocol, and sample collection followed our previous study [12]. In brief, five isoprotein diets were formulated: a basal diet containing 5% lipid (referred to as the control group, CG), a high-fat diet containing 10% lipid (referred to as the high-fat group, HF), and three different levels of betaine supplementation in the high-fat diets (referred to as the high-fat betaine group, HFB): 1 g/kg (HFB1), 4 g/kg (HFB4), and 16 g/kg (HFB16), based on dry weight. Gibel carp (initial weight of 1.65 ± 0.04 g) were randomly divided into 15 tanks (300 L, 35 fish per tank) and fed twice daily. The daily feeding amount was equivalent to 3%–4% of their body weight and was adjusted according to the weight of the fish.

The growth performance of the fish was determined at the end of the 10-week feeding trial [12]. After fast (24 hr) and anesthetization, the hepatopancreas of three fish from each tank were collected and fixed in 4% paraformaldehyde for histology and lipid deposition analysis. For gene expression analysis, the hepatopancreas samples from an additional six fish from each tank were immediately frozen in liquid nitrogen and stored at −80°C until use.

### 2.2. L8824 Cells Culture and Treatment

L8824 cells (GDC0085) derived from the hepatopancreas of grass carp were obtained from the China Center for Type Culture Collection (CCTCC, Wuhan, China). The cells were cultured in MEM medium supplemented with 10% fetal bovine serum and 1% penicillin–streptomycin. The cells were incubated at 28°C with 5% CO_2_. The growth and status of the cells were monitored regularly, and the medium was changed regularly based on cell density and status. The cells were divided into three groups: the control group (CCG) cultured in a normal medium without additional treatment, the high-fat group (CHF) exposed to a medium containing 400 *μ*mol/L of oleic acid to induce lipid accumulation, and the high-fat betaine group (CHFB) exposed to a medium containing 400 *μ*mol/L of oleic acid along with 400 *μ*mol/L of betaine.

### 2.3. Measurement of TG and VLDL

The content of TG and VLDL was evaluated using commercial kits (Nanjing Jiancheng Bioengineering Institute, China) following the manufacturer's instructions.

### 2.4. Oil Red-O Staining

The hepatopancreas samples were fixed in 4% paraformaldehyde for 30 min, rinsed with distilled water, and stained with Oil red-O for 10 min. Hematoxylin solution was used for counterstaining for 5 min before sealing with glycerin gelatin sealant. The sections were photographed under 200x magnification, and 10 images from each section were randomly obtained for integrated optical density (IOD) analysis using Image Pro Plus 6.0.

The L8824 cells were washed with PBS twice, fixed in 4% paraformaldehyde for 15 min, rinsed with PBS, and stained with Oil red-O for 30 min. The cells were photographed under 400x magnification, and 10 images from each section were obtained for IOD analysis.

### 2.5. Quantitative Real-Time PCR (qRT-PCR) Analysis

Total RNA was extracted from the hepatopancreas of gibel carp using TRIzol Reagent (Invitrogen, USA) and was treated with gDNA Eraser (Takara, Dalian, China) to remove possible DNA contaminants according to the manufacturer's instructions. qRT-PCR was conducted using a quantitative thermal cycler (Eppendorf, Germany), and the relative expression levels of target genes were determined using the 2^−*ΔΔ*CT^ method [22]. The qRT-PCR primers for *Mttp, Hnf4α*, and *Apob* genes were designed using Primer Premier 5 software (Table 1) and synthesized by Biosune (Shanghai, China).

### 2.6. Western Blot

Hepatopancreas samples were dissected into small pieces of 1–2 mm^3^ and homogenized in radioimmunoprecipitation assay (RIPA) lysis buffer (Sangon Biotech, Shanghai, China) with Halt™ Protease and Phosphatase Inhibitor Cocktail (Thermo Fisher Scientific, Waltham, MA, USA). Then, the homogenate was centrifuged at 12,000 *g* for 5 min with the supernatant collected for western blotting. Similarly, the total protein of L8824 cells was extracted using RIPA lysis buffer (Beyotime, Jiangsu, China) with protease inhibitors.

Both hepatopancreas and cell lysates were separated on SDS–PAGE, transferred to a PVDF membrane, and blocked with nonfat dry milk. Primary antibodies against HNF4*α* (1 : 1,000), MTTP (1 : 1,000), and ApoB (1 : 1,000; Abcam, USA) were incubated with the membrane, followed by incubation with second antibodies. Immunoreactive protein band intensities were analyzed using Image J software.

### 2.7. Dual-Luciferase Reporter Assay

Genomic DNAs and total RNA were extracted from the hepatopancreas using the DNA Kit (Biomarker, Beijing, China) and Total RNA Isolation Kit (Biomarker) according to the manufacturer's protocol. Specific primers targeting the promoter of *Mttp, Apob*, and the coding sequence of *Hnf4α* were designed according to the goldfish reference genome (ASM336829v1; Table 1). PCR products were then cloned into the pEASY-T1 simple cloning vector (Transgen, Beijing, China) and sequenced by Sangon Biotech (Shanghai, China).

The 293T cells (GDC0187) obtained from the China Center for Type Culture Collection (CCTCC, Wuhan, China) were maintained in DMEM supplied with 10% fetal bovine serum. Once reached 40%–50% confluence, the cells were seeded into 24-well plates and cotransfected using the Lipo2000 system (Invitrogen, Carlsbad, America) at 70% confluence. For each experiment, 300 ng of pcDNA-HNF4*α* plasmid, 180 ng of pGL3-*Mttp* or pGL3-*Apob* plasmid, and 20 ng of Renilla luciferase reporter plasmid (pRL-TK, Beyotime, China) were mixed and transfected at 26°C. PGL3-Basic and pcDNA3.1 were used as controls in the dual-luciferase reporter assay.

### 2.8. Statistical Analysis

Data were analyzed using SPSS 16.0 software, and the results were presented as the mean ± standard deviation (STD). One-way analysis of variance was conducted, followed by Tukey's multiplerange test. The level of significance was set at *P* < 0.05.

## 3. Results

### 3.1. Betaine Addition Alleviated Lipid Deposition

**Figure 1:**
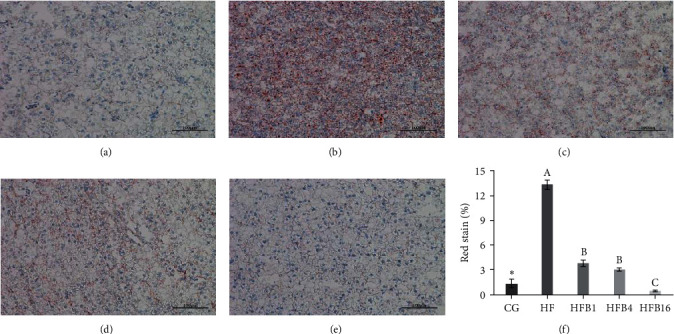
Oil red section of hepatopancreas in gibel carp: (a) CG; (b) HF; (c) HFB1; (d) HFB4; (e) HFB16; and (f) red stain of hepatopancreas in gibel carp. Different letters indicated the presence of a significant difference between the high-fat diet groups (*P* < 0.05). ^ ^*∗*^^ Significant difference compared with the HF group (*P* < 0.05).

**Figure 2:**
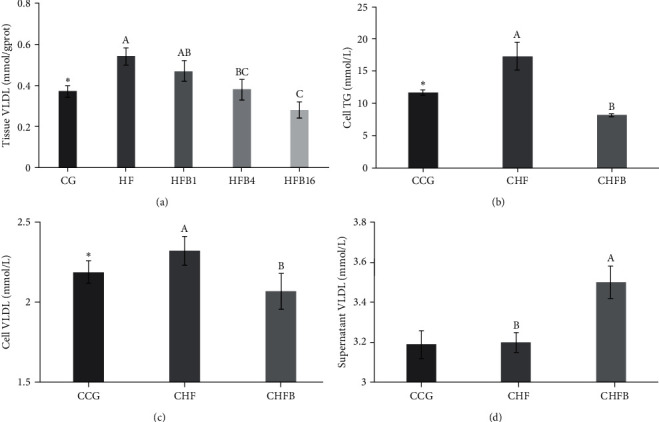
The effect of betaine addition on VLDL secretion: (a) VLDL contents in the hepatopancreas of gibel carp; (b) TG contents of L8824 cells; (c) VLDL contents of L8824 cells; and (d) VLDL contents of supernatant.  ^*∗*^ Significant difference compared with the HF group (*P* < 0.05).

Oil red-O staining experiment revealed a significant increase in the content of lipid droplets in the hepatopancreas under high-fat treatment (*P* < 0.05, Figure 1). A marked infiltration of lipids in the hepatocytes was also observed in the HF group. However, when betaine was supplemented, the content of lipid droplets decreased in a dose-dependent manner (*P* < 0.05, Figure 1). Interestingly, the content of VLDL was also affected by high-fat treatment and dietary betaine supplementation similarly (*P* < 0.05, Figure 2(a)).

**Figure 3:**
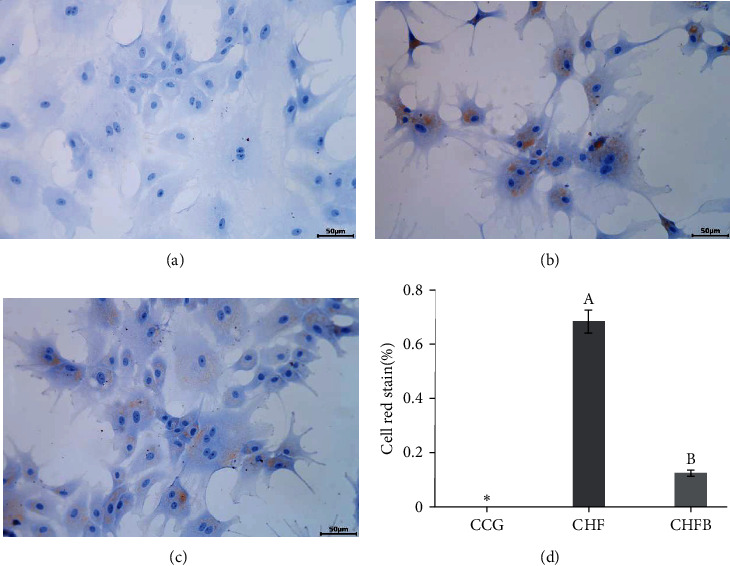
Oil red section of L8824 cells: (a) CCG; (b) CHF; (c) CHFB; and (d) red stain of L8824 cells.  ^*∗*^ Significant difference compared with the HF group (*P* < 0.05).

Further investigation in L8824 cells showed that lipid infiltration in the CHFB group was effectively alleviated compared to the CHF group (*P* < 0.05, Figure 3). Additionally, the content of TG and VLDL were both significantly decreased in the CHFB group compared to the CHF group (*P* < 0.05, Figures 2(b) and 2(c)). On the other hand, the VLDL content in the supernatant of the CHFB group was significantly enhanced compared to the CHF group (*P* < 0.05, Figure 2(d)).

### 3.2. Gene Expression in Response to Dietary Betaine

**Figure 4:**
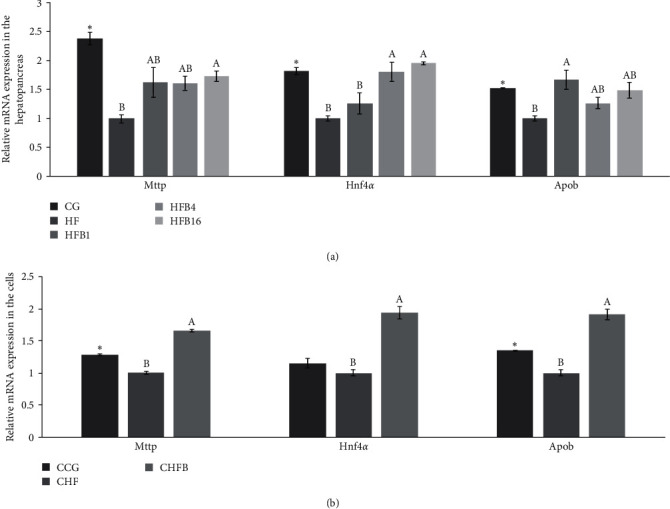
Expression of mRNAs for Mttp, Hnf4*α*, and Apob in: (a) hepatopancreas and (b) L8824 cells.  ^*∗*^ Significant difference compared with the HF group (*P* < 0.05).

High-fat treatment significantly reduced the mRNA expression levels of *Mttp* and *Apob* both *in vivo* and *in vitro* compared to the control group (CG, and CCG; *P* < 0.05, Figure 4). Similarly, the high-fat diet significantly reduced the expression level of *Hnf4α* in the hepatopancreas (*P* < 0.05, Figure 4). However, adding betaine significantly increased the mRNA expression levels of *Hnf4α, Mttp*, and *Apob* both *in vivo* and *in vitro* (*P* < 0.05, Figure 4).

**Figure 5:**
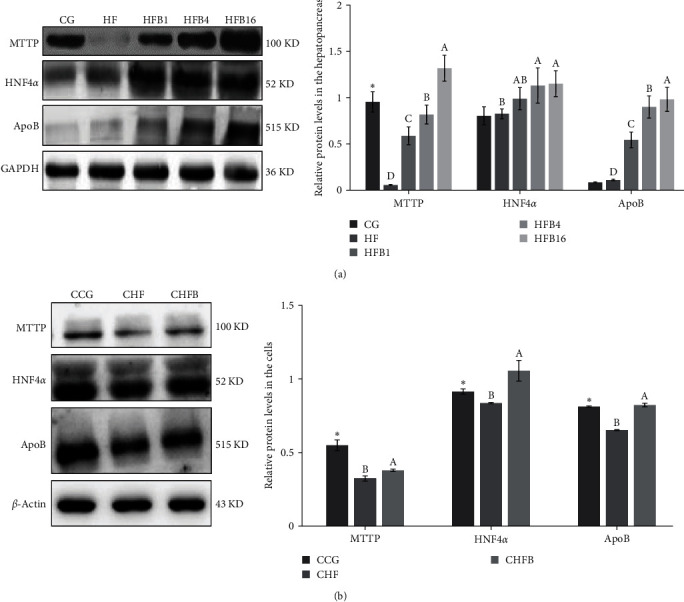
Total protein abundances for MTTP, HNF4*α*, and ApoB in: (a) hepatopancreas and (b) L8824 cells.  ^*∗*^ Significant difference compared with the HF group (*P* < 0.05).

The protein levels of these three genes were also significantly affected by high-fat treatment and betaine supplementation. For example, high-fat treatment significantly downregulated the protein level of MTTP compared to the CG (or CCG) group (*P* < 0.05, Figure 5). As for HNF4*α* and ApoB, downregulation was only observed in the cells. Under betaine treatment, however, the protein expression levels of HNF4*α*, MTTP, and ApoB were significantly upregulated *in vivo* and *in vitro* (*P*  < 0.05, Figure 5).

### 3.3. Promoter Activity in 293T Cell Line

**Figure 6:**
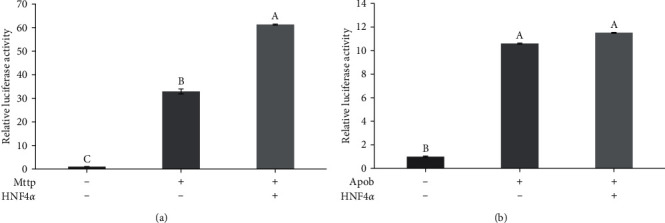
Dual-luciferase detection results: (a) Mttp and (b) Apob. “+” means present in the system, on the contrary “−” means absent. Luciferase activity is presented as the mean ± SEM (*n* = 3). Different letters indicated the presence of a significant difference between the groups (*P* < 0.05).

Here we show that both promoters of *Mttp* and *Apob* of gibel carp initialized significantly higher marker gene expression levels than the PGL3-Basic control group (*P* < 0.05, Figure 6), indicating that the two promoters were active and capable of driving gene expression in the 293T cells. Moreover, the introduction of the HNF4*α* expression plasmid led to an approximately 2-fold upregulation of the *Mttp* promoter activity (*P* < 0.05, Figure 6(a)). However, no significant difference was observed in the promoter activity of *Apob* with the introduction of HNF4*α* (*P*  > 0.05, Figure 6(b)).

## 4. Discussion

It is well-established that prolonged consumption of high-fat diets can lead to metabolic disorders and adversely affect the growth and health of farmed fish [6, 23]. Research has shown that betaine, as a feed additive, exhibits attractive properties and affects fat distribution in various fish species [11, 24, 25].

In this study, we employed the gibel carp as a model to investigate lipid metabolism in the context of a high-fat diet challenge. Our findings demonstrated that a 10-week period of high-fat feeding significantly increased TG levels in both the serum and hepatopancreas of gibel carp, consistent with similar studies conducted in other fish species [2, 6, 23]. However, when betaine was added to the high-fat diet, the TG content decreased significantly compared to the high-fat feeding group, suggesting a strong effect of betaine in reducing lipid deposition. Similar effects have been observed in other fish species.

Considering the crucial role of VLDL in hepatic lipid metabolism, we further focused on the impact of high-fat feeding and betaine supplementation on VLDL secretion. Our results revealed that high-fat feeding increased VLDL content in the hepatopancreas of gibel carp and L8824 cells, while betaine addition reduced both. Notably, the secretion of VLDL was significantly enhanced by betaine treatment, as indicated by the higher VLDL content in the cell liquid supernatant compared to the control (CHF) group. This observation was consistent with studies conducted in mammals, where betaine enhances TG secretion through VLDL production and release, counteracting the reduction caused by a high-fat diet [6, 26]. Thus, we speculated that high-fat treatment suppressed the synthesis and secretion of VLDL, while the addition of betaine alleviated this suppression.

To validate this hypothesis, we further compared the expression levels of key genes involved in VLDL secretion and regulation under high-fat feeding conditions with and without betaine treatment. MTTP and ApoB are key molecules involved in VLDL assembly and secretion, where MTTP facilitates the translocation of ApoB mRNA across the endoplasmic reticulum and transfers newly synthesized TG to ApoB during VLDL assembly [27]. In our study, we observed a significant decrease in the relative protein levels of MTTP, and ApoB in L8824 cells subjected to high-fat treatment. However, betaine addition reversed the inhibition effect induced by the high-fat diet. Thus, it is evident that a high-fat diet and betaine addition regulate hepatic lipid deposition in gibel carp via suppression and improvement of VLDL secretion capacity, respectively.

Given that HNF4*α* acts as a transcriptional activator of the *Mttp* and *Apob* genes in mammals [28, 29], we further explored the effect of betaine addition on HNF4*α* expression in L8824 cells. We found that the protein level of HNF4*α* was also affected by high-fat treatment and betaine supplementation in the same way as MTTP and ApoB. Furthermore, we found that HNF4*α* upregulated the promoter activity of *Mttp* in gibel carp, leading to improved VLDL secretion, which is consistent with findings on the mechanism of *Mttp* inhibition, where HNF4*α* significantly enhanced the promoter activity of *Mttp* [30]. In contrast, HNF4*α* showed no regulatory activity on the *Apob* promoter, which might reflect distinct mechanisms of *Apob* expression regulation between mammals and fishes. Disruption of *Mttp* and *Apob* expression by high-fat feeding has also been reported in other fish species [2], which was probably associated with reduced promoter activity due to decreased *Hnf4α* expression.

Based on these findings, it can be inferred that the HNF4*α*/MTTP pathway is involved in betaine regulating lipid metabolism in hepatocytes of fish via controlling VLDL secretion. Further research on the mechanism through which dietary betaine regulates the expression of *Hnf4α, Mttp*, and *Apob* will shed light on identifying new dietary additives that help alleviate excessive lipid deposition in the context of high-fat feeding.

## Figures and Tables

**Table 1:** Information of primers used in this study.

Primers^1^	Sequence 5′−3′ ^2^	Purpose	Efficiency (%)
H-MTTP forward	TTATGAGCAGTAGTCCGTCTT	Hepatopancreas qRT-PCR	104.52
H-MTTP reverse	GACCCAGTCTTTGAGAACCT		
H-HNF4*α* forward	ATCCCAGCATTCTGTGACCTT	Hepatopancreas qRT-PCR	98.69
H-HNF4*α* reverse	TGGAAGGGAAGCACTAACTCAT		
H-ApoB forward	AGCAGCAACTTCTACAACT	Hepatopancreas qRT-PCR	103.14
H-ApoB reverse	TTATGGATAGGAGGTCAACAG		
H-EF-1*α* forward	CGCCAGTGTTGCCTTCGT	Hepatopancreas qRT-PCR	99.86
H-EF-1*α* reverse	CGCTCAATCTTCCATCCCTT		
C-MTTP forward	TCAGGATACCACTATGGAGTTCAC	L8824 cell qRT-PCR	98.61
C-MTTP reverse	TACAGCGGCGTTCACACTTG		
C-HNF4*α* forward	TGGTGCTGGTGGAATGG	L8824 cell qRT-PCR	99.08
C-HNF4*α* reverse	ATCTTGGAAGGGAAGGACTAA		
C-ApoB forward	CGTATTGCTGCCTATCTCATT	L8824 cell qRT-PCR	100.96
C-ApoB reverse	CTTAGTTTCTGGTGCTGTGGA		
C-*β*-actin forward	AAGTATCCTATTGAGCACGGTATTG	L8824 cell qRT-PCR	100.44
C-*β*-actin reverse	TCAGGGGAGCCTCTGTAAGC		
L-MTTP forward	TAGGTACCCAAGATAAGCAGCATCAACACCA	Dual-luciferase assay	—
L-MTTP reverse	CCAAGCTTGGTTGGCTCGCTGAGAGCTGTG		
L-ApoB forward	TAGGTACCCTAATCACTGTGTTTTAT	Dual-luciferase assay	—
L-ApoB reverse	CCAAGCTTCGTTATGGGCACTGAAACTTC		
L-HNF4a forward	CCGGAATTCATGCGTTTGTCCAAACCAC	Dual-luciferase assay	—
L-HNF4a reverse	CCGCTCGAGTCAGATGGCCTCTTGTTTGG		

^1^The prefix of the primer name indicates the purpose of the primer. H, Hepatopancreas; C, L8824 cell; L, Dual-luciferase assay. ^2^The underscored bases represent the recognition sequence of a restriction endonuclease.

## Data Availability

The data used to support the findings of this study are available from the first author (xjdong@yzu.edu.cn) upon request.
